# Teamwork and implementation of innovations in healthcare and human service settings: a systematic review

**DOI:** 10.1186/s13012-024-01381-9

**Published:** 2024-07-15

**Authors:** Elizabeth A. McGuier, David J. Kolko, Gregory A. Aarons, Allison Schachter, Mary Lou Klem, Matthew A. Diabes, Laurie R. Weingart, Eduardo Salas, Courtney Benjamin Wolk

**Affiliations:** 1 Department of Psychiatry, School of Medicine, University of Pittsburgh, 3811 O’Hara Street, Pittsburgh, PA 15213 USA; 2 Department of Psychiatry, University of California San Diego, La Jolla, CA USA; 3 ACTRI Dissemination and Implementation Science Center, UC San Diego, La Jolla, CA USA; 4 Child and Adolescent Services Research Center, San Diego, CA USA; 5 Department of Psychiatry, Perelman School of Medicine, University of Pennsylvania, Philadelphia, PA USA; 6 Penn Implementation Science Center at the Leonard Davis Institute of Health Economics, University of Pennsylvania, Philadelphia, PA USA; 7 Health Sciences Library System, University of Pittsburgh, Pittsburgh, PA USA; 8 Tepper School of Business, Carnegie Mellon University, Pittsburgh, PA USA; 9 Department of Psychological Sciences, Rice University, Houston, TX USA

**Keywords:** Team, Teamwork, Implementation outcomes, Systematic review

## Abstract

**Background:**

Implementation of new practices in team-based settings requires teams to work together to respond to new demands and changing expectations. However, team constructs and team-based implementation approaches have received little attention in the implementation science literature. This systematic review summarizes empirical research examining associations between teamwork and implementation outcomes when evidence-based practices and other innovations are implemented in healthcare and human service settings.

**Methods:**

We searched MEDLINE, CINAHL, APA PsycINFO and ERIC for peer-reviewed empirical articles published from January 2000 to March 2022. Additional articles were identified by searches of reference lists and a cited reference search for included articles (completed in February 2023). We selected studies using quantitative, qualitative, or mixed methods to examine associations between team constructs and implementation outcomes in healthcare and human service settings. We used the Mixed Methods Appraisal Tool to assess methodological quality/risk of bias and conducted a narrative synthesis of included studies. GRADE and GRADE-CERQual were used to assess the strength of the body of evidence.

**Results:**

Searches identified 10,489 results. After review, 58 articles representing 55 studies were included. Relevant studies increased over time; 71% of articles were published after 2016. We were unable to generate estimates of effects for any quantitative associations because of very limited overlap in the reported associations between team variables and implementation outcomes. Qualitative findings with high confidence were: 1) Staffing shortages and turnover hinder implementation; 2) Adaptive team functioning (i.e., positive affective states, effective behavior processes, shared cognitive states) facilitates implementation and is associated with better implementation outcomes; Problems in team functioning (i.e., negative affective states, problematic behavioral processes, lack of shared cognitive states) act as barriers to implementation and are associated with poor implementation outcomes; and 3) Open, ongoing, and effective communication within teams facilitates implementation of new practices; poor communication is a barrier.

**Conclusions:**

Teamwork matters for implementation. However, both team constructs and implementation outcomes were often poorly specified, and there was little overlap of team constructs and implementation outcomes studied in quantitative studies. Greater specificity and rigor are needed to understand how teamwork influences implementation processes and outcomes. We provide recommendations for improving the conceptualization, description, assessment, analysis, and interpretation of research on teams implementing innovations.

**Trial registration:**

This systematic review was registered in PROSPERO, the international prospective register of systematic reviews. Registration number: CRD42020220168.

**Supplementary Information:**

The online version contains supplementary material available at 10.1186/s13012-024-01381-9.

Contributions to the Literature:

- This paper reviews more than 20 years of research on teams and implementation of new practices in healthcare and human service settings.
- We concluded with high confidence that adaptive team functioning is associated with better implementation outcomes and problems in team functioning are associated with poorer implementation outcomes. While not surprising, the implementation science literature has lacked clear empirical evidence for this finding.
- Use of the provided recommendations will improve the quality of future research on teams and implementation of evidence-based practices.

## Background

Healthcare and human service providers (e.g., clinicians, case managers) often work in team-based settings where professionals work collaboratively with one another and service recipients toward shared goals [1, 2]. Team-based care is intended to include multiple professionals with varying skills and expertise [1, 3]. It requires shared responsibility for outcomes and increases team members’ dependence on one another to complete work [1, 3, 4]. Effective team-based care and higher quality teamwork are associated with improvements in care access and quality, patient safety, patient satisfaction, clinical outcomes, and costs [2, 4–9].

**Fig. 1:**
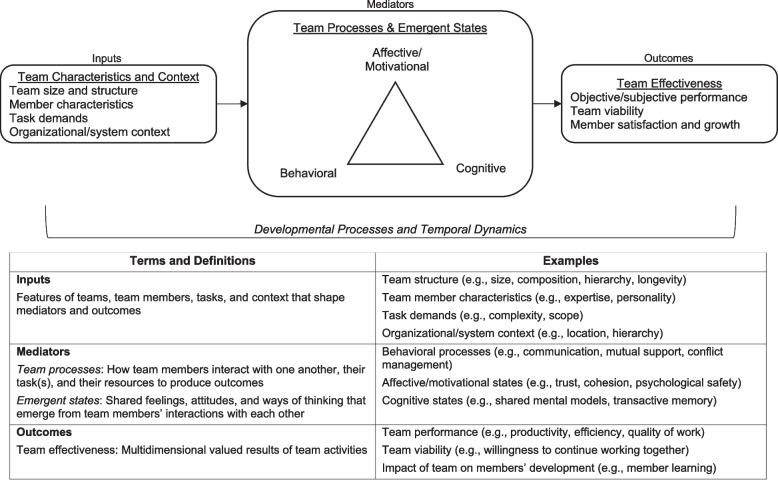
Conceptual model of team effectiveness and key terminology. Figure adapted from “Advancing research on teams and team effectiveness in implementation science: An application of the Exploration, Preparation, Implementation, Sustainment (EPIS) framework” by E.A. McGuier, D.J. Kolko, N.A. Stadnick, L. Brookman-Frazee, C.B. Wolk, C.T. Yuan, C.S. Burke, & G.A. Aarons, 2023, *Implementation Research and Practice*, *4*, 26334895231190855. [CC BY-NC]

We use the term ‘teamwork’ to refer to an array of team constructs using the input-mediator-outcome-input (IMOI) framework (Fig. 1) [10–12]. The IMOI framework recognizes that team interactions are dynamic and complex, with processes unfolding over time and feedback loops between processes, outcomes, and inputs [10]. Team inputs include team structure and composition, task demands, and contextual features [13]. Mediators are aspects of team functioning (i.e., what team members think, feel, and do [12]) through which inputs influence outcomes. These processes and emergent states may be cognitive, affective, or behavioral [5, 14–16]. Team effectiveness outcomes are multidimensional and include team performance as well as team viability and the impact of the team on members’ development [12, 17–19].

Implementation of new practices in team-based service settings requires team members to work together to respond to changing demands and expectations. Extensive research has identified barriers and facilitators to implementation of new practices at the individual provider, organization, and system levels; however, the team level has received little empirical attention [20, 21]. This is a problem because implementation efforts increasingly rely on teams, and responses to a new practice are likely to be influenced by team characteristics and processes. See McGuier and colleagues [20] for an overview of team constructs in the context of implementation science and the Exploration, Preparation, Implementation, Sustainment (EPIS) framework [22, 23]. Given increasing use of team-based care and interest in implementation strategies targeting teams, examining how teamwork is associated with implementation processes and outcomes is critical. This systematic review identified and summarized empirical research examining associations between teamwork and implementation outcomes when evidence-based practices (EBPs) and other innovations were implemented in healthcare and human service settings.

## Methods

This systematic review was registered (PROSPERO; registration number: CRD42020220168) and conducted following the published protocol [24]. The review was conducted in accordance with PRISMA and SWiM guidance [25, 26]; relevant checklists are in Additional File 1.

### Information sources and search strategy

We searched the following databases: MEDLINE (Ovid), CINAHL (Ebsco), APA PsycINFO (Ovid), and ERIC (Ebsco). Database searches were run on August 7, 2020, and again on March 8, 2022. For all searches, a publication date from 2000 to current was applied; there were no language restrictions (see [24]). An experienced health sciences librarian (MLK) designed the Ovid MEDLINE search and translated that search for use in the other databases (see additional file in [24]). The search strings consisted of controlled vocabulary (when available) and natural language terms representing concepts of teamwork and implementation science or innovation or evidence-based practice. Results were downloaded to an EndNote (version X9.3.3) library and duplicate records removed [27]. Additional relevant articles were identified by hand searches of reference lists of included articles, a cited reference search for included articles in the Web of Science (Clarivate) bibliographic database (completed in February 2023), and requests sent to implementation science listservs and centers for suggestions of relevant articles.

### Eligibility criteria

We included empirical journal articles describing studies using quantitative, qualitative, or mixed methods. Study protocols, reviews, and commentaries were excluded. All studies were conducted in healthcare or human service settings (e.g., hospitals, clinics, child welfare) and described the implementation of a practice to improve patient care. Studies of interventions to improve teamwork (e.g., team building interventions) and studies of teams created to implement the innovation (e.g., quality improvement teams, implementation support teams) were excluded. Eligible studies assessed at least one team construct and described its influence on implementation processes and outcomes.

### Changes from protocol

Several changes were made from our systematic review protocol (PROSPERO CRD42020220168; [24]). Specifically, during the full-text review stage, we broadened the scope from team functioning (i.e., processes and states) to include team structure and performance because of the small number of studies that assessed and reported specific processes or states. This change increased the number of included studies. Similarly, because implementation outcomes were often inconsistently defined and poorly reported [28–30], we broadened our scope to include studies that identified team constructs as implementation determinants (i.e., barriers/facilitators) without explicitly defining and measuring an implementation outcome. Because of changes in university access to bibliographic databases, the cited reference search was performed in the Web of Science only instead of the Web of Science and Scopus. This bibliographic database indexes more than 21,000 scientific journals [31]. Lastly, because of time and resource constraints, we did not search conference abstracts or contact authors for unreported data.

### Selection process and data extraction

Title/abstract screening and review of full-text articles were conducted by pairs of trained independent reviewers in DistillerSR. Conflicts were resolved through re-review, discussion between reviewers, and when needed, discussion with a senior team member (EAM). A final review of all included articles was conducted by EAM. Relevant data from each article was extracted into an Excel spreadsheet by one reviewer (AS). A second reviewer (EAM) conducted a line-by-line review and verification. Our data extraction form was informed by existing forms and guides (e.g., [32, 33]). For each included study, we extracted information on measures of teamwork and implementation-relevant outcomes, characteristics of the setting, teams, and participants, analysis methods, and results. For quantitative studies, we recorded correlation coefficients and/or regression coefficients as standardized metrics of association. For qualitative studies, we recorded themes [33].

### Quality and risk of bias assessment

The Mixed Methods Appraisal Tool (MMAT) [34] was used to evaluate quality and risk of bias for each included study. Multiple publications from the same study were evaluated separately because they reported different outcomes. Consistent with Powell and colleagues [35], quality evaluations were only made for the components of the study relevant to our question. Quality evaluations were conducted by two independent reviewers (EAM, MAD) with discrepancies resolved through consensus discussion. After completing the MMAT, the reviewers jointly categorized each article as high, moderate, or low quality. High quality studies were those with affirmative responses to all MMAT questions. Moderate quality studies had at least one minor methodological problem, and low-quality studies had serious flaws (e.g., qualitative studies with poor coherence between data, analysis, and conclusions; quantitative studies with biased samples and/or inappropriate statistical analyses).

We rated the relevance of each publication to our research question as high, moderate, or low. Highly relevant studies reported implementation of a well-defined innovation, thoroughly described team constructs and implementation outcomes, and clearly linked team constructs to implementation outcomes. Most studies rated as low relevance provided very limited information about teamwork and/or implementation outcomes. Studies that only described barriers/facilitators were rated as low or moderate relevance. Ratings were conducted by two independent reviewers (EAM, CBW) with discrepancies resolved through consensus discussion.

### Data synthesis

We conducted a narrative synthesis of included studies following guidelines for synthesis without meta-analysis (SWiM) [36]. We prioritized reporting of high quality, highly relevant studies. Studies categorized as low quality and/or low relevance were not included in the synthesis but are included in the description of study characteristics to convey the breadth of the literature. We organized studies based on the IMOI framework (i.e., team inputs, processes/states, and outputs) and organized studies of processes/states by affective, behavioral, and cognitive constructs when possible. Because of the heterogeneity in team constructs and implementation outcomes, we were unable to quantitatively synthesize results using meta-analysis or formally investigate heterogeneity; this challenge is common in implementation science systematic reviews [30]. We assessed the strength of the overall body of evidence with GRADE for quantitative studies [37] and GRADE-CERQual for qualitative studies [38, 39]. GRADE results in ratings of high, moderate, low, or very low quality of evidence for each outcome of interest. GRADE-CERQual results in ratings of high, moderate, low, or very low confidence in each review finding. GRADE ratings were made independently with discrepancies resolved through consensus discussion; GRADE-CERQual ratings were made through iterative discussions as recommended [39]. All ratings and decisions were made by the first and senior authors.

## Results

### Search results

**Fig. 2:**
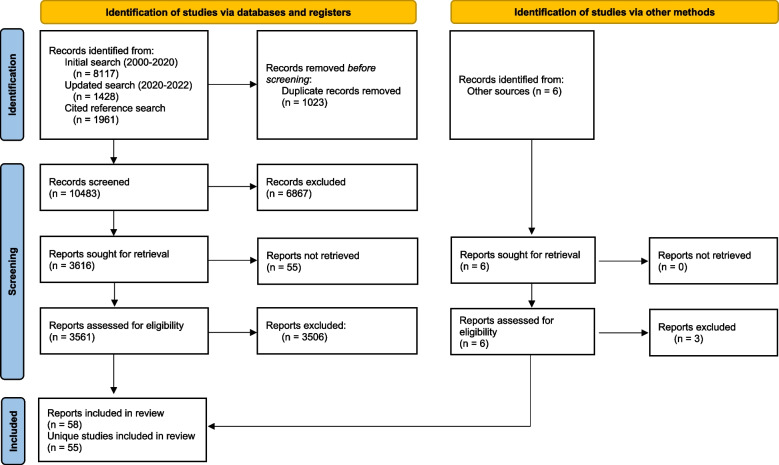
PRISMA flow diagram of included articles. *From:* Page MJ, McKenzie JE, Bossuyt PM, Boutron I, Hoffmann TC, Mulrow CD, et al. The PRISMA 2020 statement: an updated guideline for reporting systematic reviews. BMJ 2021;372:n71. https://doi.org/10.1136/bmj.n71. For more information, visit: http://www.prisma-statement.org/

Our initial search, after removal of duplicates, yielded 7181 results. The second search (August 2020-March 2022) captured an additional 1341 results. The cited reference search yielded 1961 results. A total of 10,489 results were included in title/abstract review. Figure 2 provides a PRISMA flow diagram for included studies. After full-text review, 58 articles from 55 studies were included in analyses [40–97].

**Fig. 3:**
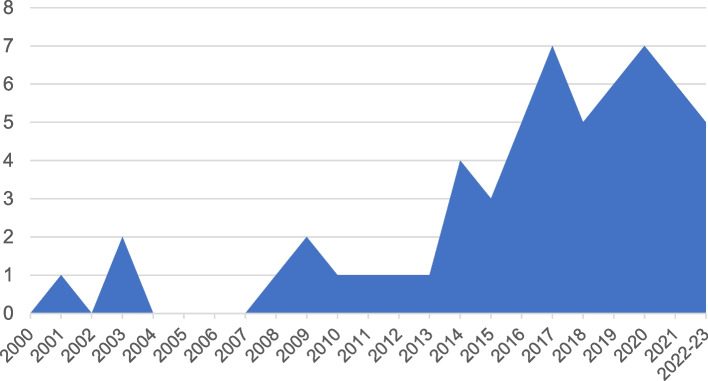
Included articles by year of publication

As shown in Fig. 3, publications on teamwork and implementation have increased substantially since 2000. Three articles on this topic (5%) were published between 2000 and 2007, 14 (24%) between 2008 and 2015, and 41 (71%) between 2016 and early 2023.

### Study characteristics

Interrater agreement was good for assessment of study quality (81% agreement on MMAT questions) and ratings of relevance (88% agreement). There were 20 high quality articles, 23 moderate quality articles, and 15 low quality articles. Fourteen articles were rated as high relevance, 22 as moderate, and 22 as low relevance. Only 4 were rated as both high quality and high relevance. We report study characteristics for all 58 eligible articles. Our narrative synthesis includes 32 articles categorized as moderate/high quality and moderate/high relevance; it excludes 26 articles categorized as low quality and/or relevance.

Studies were conducted in inpatient healthcare (*n* = 22), outpatient/ambulatory healthcare (*n* = 21), mental health settings (*n* = 9), and other settings (e.g., residential facilities, multiple settings; *n* = 6). There were 33 qualitative, 15 quantitative, and 10 mixed methods studies. All quantitative studies were descriptive observational studies.

Most studies examined team processes/states (*n* = 53); fewer examined team inputs (*n* = 27). Only two studies examined a team effectiveness outcome. The most common implementation outcomes were fidelity (*n* = 16) and other specified implementation outcomes (e.g., “extent of use,” “implementation success”) (*n* = 15). Less frequently identified implementation outcomes included adoption (*n* = 5), sustainment (*n* = 4), reach (*n* = 4), and perceptions of the innovation (e.g., acceptability, appropriateness, feasibility; *n* = 3). Approximately one-third of studies (*n* = 21) did not report specific implementation outcomes but described implementation determinants (i.e., barriers and facilitators).

### Synthesis: team inputs & implementation outcomes

Team inputs examined in studies included team stability/instability and staffing shortages, aspects of team structure and composition, interdependence, and hierarchy and professional roles. Quantitative findings are presented in Table 1. A CERQual Summary of Qualitative Findings related to team inputs is shown in Table 2. A CERQual Evidence Profile is provided in Additional File 2 (Table A1).

**Table 1:** Summary of quantitative studies: team inputs & implementation outcomes

*Team Input*	*Implementation Outcome*					
	**Acceptability, appropriateness, feasibility**	**Adoption**	**Fidelity**	**Reach**	**Sustainment**	**Other**
**Stability**						*r* = 0.18, ns (implementation success) [48]
**Team size**		B = 0.01, ns [72]	***r* = 0.28** [47]	**B = -0.21** [72]		
**Workload**					***r* = -0.54** [68]	
**Longevity**			*r* = 0.10, ns [47]			
**History of change**					*r* = -0.02, ns [68]	
**Task interdependence**	*r* = 0.09, ns (acceptability) *r* = 0.06, ns (appropriateness) *r* = 0.08, ns (feasibility) [65]	*r* = -0.32, ns [65]		***r* = 0.60** (initial reach) ***r* = 0.55** (overall reach) [65]		
**Outcome interdependence**	*r* = 0.04, ns (acceptability) *r* = 0.04, ns (appropriateness) *r* = 0.06, ns (feasibility) [65]	***r* = -0.52** [65]		*r* = 0.01, ns (initial reach) *r* = 0.12, ns (overall reach) [65]		

Low quality/low relevance studies not included in synthesis: [44, 57, 58, 89]

Bolded results are statistically significant

*ns* Not statistically significant

**Table 2:** Summary of qualitative findings: team inputs & implementation outcomes

Summary of Review Finding	Studies Contributing	CERQual Assessment of Confidence in the Evidence	Explanation of CERQual Assessment
Dedicated and stable team members facilitate implementation, while instability in team membership is a barrier to implementation	[49, 70, 81, 94]	Moderate confidence	Only 4 studies with limited diversity in settings and interventions leading to moderate concerns about adequacy and relevance
Staffing shortages and turnover hinder implementation	[50, 67, 75, 78, 92]	High confidence	Minor concerns about methodology and adequacy do not reduce confidence in this simple descriptive finding
Team member competency/expertise, experience, and commitment/engagement facilitate implementation	[40, 70, 81, 84, 95]	Moderate confidence	Only 5 studies with variation across studies and thin data leading to moderate concerns about adequacy and coherence
In multidisciplinary settings, rigid professional roles, hierarchical relationships, and power differentials are barriers to implementation	[50, 53, 74, 97]	Moderate confidence	Only 4 studies with variation across studies and thin data leading to moderate concerns about coherence and adequacy

Low quality/low relevance studies not included in synthesis: [46, 51, 60, 69, 73, 76, 79, 87, 89]

#### Team stability/instability and staffing shortages

Team stability/instability (i.e., consistency in membership over time) was examined in one mixed methods study [48, 49] and three qualitative studies [70, 81, 94]. A study of surgical teams found variations in membership stability but no association between stability and “implementation success” (i.e., composite measure based on number of uses of new technique, proportion of uses, and changes in use) [48, 49]. The authors suggested that stability facilitates the development of team coordination but that selecting small and exclusive teams may limit the spread of innovations within the organization. Another study found that a dedicated and stable team in which members were selected and trained together in the use of a new surgical technique led to quicker uptake and better integration into practice, theorizing that dedicated and stable teams increased trust, motivation, and collaborative problem-solving [81]. However, dedicated teams were difficult to sustain, and some sites instead used rotating team members from a larger pool of trained staff. In rural primary care, stability of team members facilitated sustainment of memory care clinics [70]. Lastly, another study in primary care found mixed perceptions of stable vs. rotating staff when adding a new team role (i.e., health coach); some team members liked rotating through different roles while others wanted more stability [94]. Across studies, we found that dedicated and stable team members facilitate implementation while instability in team membership is a barrier to implementation (moderate confidence).

Qualitative studies identified staffing shortages and turnover on teams as barriers to implementation [50, 67, 75, 78, 92]. In Veterans Health Administration (VA) clinics, “inadequate staffing posed an insurmountable barrier,” hindering communication and delivery of optimal care during the implementation of the patient-centered medical home (PCMH) model [92]. Similarly, staff shortages, turnover, and high workloads hindered guideline implementation in Kenyan hospitals [75]. Two studies found negative impacts of staffing shortages and turnover on sustainment. Staff turnover contributed to discontinuity in Dialectical Behavior Therapy (DBT) team members [78], and appropriate staffing (i.e., ensuring manageable workloads) and blocking time for team members were identified as critical to sustainment of a team-based model in the VA [67]. We found that staffing shortages and turnover hinder implementation (high confidence).

#### Team structure/composition

Studies examined multiple aspects of team structure and composition, specifically team size, workload, longevity (i.e., how long team members had worked together), history of change, and team member characteristics. Team size was examined in two studies of DBT. In a mixed methods study, team size was positively correlated with fidelity, and qualitative data suggested that team size may increase as a result of successful implementation [47]. In contrast, another study found that DBT team size was not associated with the number of DBT components adopted and was negatively associated with reach, suggesting reach may reflect high workloads [72]. In VA mental health clinics, team workload (i.e., number of patients seen) was negatively associated with sustainment of trauma-focused therapies [68]. In these studies, team longevity and history of change were not associated with implementation outcomes [47, 68]. Team member characteristics, specifically team member competency/expertise, experience, and commitment/engagement, were identified as facilitators of implementation in some qualitative studies [40, 70, 81, 84, 95].

Overall, few findings could be made from quantitative studies examining team structure and composition. Two studies of team size found mixed results, and workload, longevity, and history of change were examined in only one study each. Across qualitative studies, we found team member competency/expertise, experience, and commitment/engagement facilitate implementation (moderate confidence).

#### Team interdependence

One quantitative study examined team interdependence [65]. In multidisciplinary child abuse teams implementing a mental health screening/referral protocol, task interdependence (i.e., reliance on team members to share resources and coordinate workflows) was positively associated with reach but not time to adoption. Outcome interdependence (i.e., extent to which outcomes are evaluated at the team vs. individual level) was significantly negatively correlated with time to adoption but not reach. Neither task nor outcome interdependence were associated with team members’ perceptions of acceptability, appropriateness, or feasibility of the innovation [65]. Because only one study examined interdependence, no review findings were made.

#### Hierarchy & professional roles

Hierarchy, power distributions, and rigid roles were identified as barriers to implementation in several qualitative studies [50, 53, 74, 97]. Flatter hierarchies (i.e., more equal distribution of power and authority) supported guideline implementation in pediatric primary care; practices with low compliance to guidelines had more hierarchical relationships while practices with high compliance had more shared decision-making [97]. In a setting with hierarchy and rigid division of roles, nurses trained in an innovation reported concern that their decisions would be questioned by physicians without expertise in the innovation but more authority [74]. Similarly, in surgical teams, rigid professional roles and a hierarchical team culture constrained open discussion and created contention over how and when a “time-out” should be completed, resulting in inconsistent use and poor fidelity [50, 53]. Across studies, we found that in multidisciplinary settings, rigid professional roles, hierarchical relationships, and power differentials are barriers to implementation (moderate confidence).

#### Summary of team inputs & implementation outcomes

There was no overlap among team input variables and implementation outcomes examined in quantitative studies (Table 1). Accordingly, we were unable to generate estimates of effects or ratings of evidence quality. Qualitative review findings are shown in Table 2. We found: 1) Dedicated and stable team members facilitate implementation while instability in team membership is a barrier to implementation (moderate confidence); 2) Staffing shortages and turnover hinder implementation (high confidence); 3) Team member competency/expertise, experience, and commitment/engagement facilitate implementation (moderate confidence); and 4) In multidisciplinary settings, rigid professional roles, hierarchical relationships, and power differentials are barriers to implementation (moderate confidence).

### Synthesis: team processes/states & implementation outcomes

Studies examined overall team functioning as well as specific affective states, behavioral processes, and cognitive states. Quantitative findings are presented in Table 3, and a GRADE Evidence Profile is provided in Additional File 2 (Table A2). A CERQual Summary of Qualitative Findings related to team processes and states is shown in Table 4. A CERQual Evidence Profile is provided in Additional File 2 (Table A3).

**Table 3:** Summary of quantitative studies of team processes/states & implementation outcomes

*Team Process/ State*	*Implementation Outcome*					
	**Acceptability, appropriateness, feasibility**	**Adoption**	**Fidelity**	**Reach**	**Sustainment**	**Other**
**Overall Team Functioning**		Odds ratio = 1.106, ns [59] B = -0.11, ns (positive functioning) B = -0.04, ns (negative functioning) [72] NA [83]	**Beta = 0.44** (baseline) **Beta = 0.79** (change over time) [45] ***r* = 0.58** [47] NA [77]	B = 0.42, ns (positive functioning) **B = 0.69** (negative functioning) [72]	***r* = .53** [68]	***r* = .43** [62] NA [88]
**Affective States**						
* Liking/trust/respect*	***r* = 0.19** (acceptability) ***r* = 0.17** (appropriateness) ***r* = 0.18** (feasibility) [65]	*r* = -0.05, ns [65]		*r* = -0.20, ns (initial reach) *r* = 0.07, ns (overall reach) [65]		
* Cohesion*			***r* = 0.43** [47]			
* Psychological safety and ease of speaking up*						***r* = 0.55** (implementation success, interviewer rating) ***r* = 0.47** (implementation success, coders’ rating) [48]
**Behavioral Processes**						
* Communication*			***r* = 0.49** [47]			
* Learning behavior and boundary spanning*	***r* = 0.10** (acceptability) *r* = 0.08, ns (appropriateness) ***r* = 0.11** (feasibility) [65]	*r* = -0.38, ns [65]		*r* = 0.03, ns (initial reach) *r* = -0.03, ns (overall reach) [65]		***r* = 0.66** (implementation success) [48] ***r* = 0.55** (use of radical innovation) ***r* = 0.68** (use of incremental innovation) [91]
**Cognitive States**						
* Shared goals*	*r* = 0.08, ns (acceptability) *r* = 0.09, ns (appropriateness) *r* = 0.08, ns (feasibility) [65]	*r* = -0.41, ns [65]		*r* = 0.04, ns (initial reach) *r* = -0.00, ns (overall reach) [65]		
* Knowledge/skill*						***r* = 0.52** [62]
* Problem recognition*						*r* = 0.14, ns [62]

Low quality/low relevance studies not included in synthesis: [42, 43, 51, 54, 55, 66, 80]

*NA* Not available (no correlation or regression coefficients reported)

Bolded results are statistically significant

*ns* Not statistically significant

**Table 4:** Summary of qualitative findings: team processes/states & implementation outcomes

Summary of Review Finding	Studies Contributing	CERQual Assessment of Confidence in the Evidence	Explanation of CERQual Assessment
Adaptive team functioning, characterized by positive affective states (e.g., trust, mutual respect, belonging), effective behavior processes (e.g., frequent communication and coordination), and shared cognitive states (e.g., clear roles, shared mental models of how to provide care), facilitates implementation and is associated with better implementation outcomes Problems in team functioning, including negative affective states (e.g., tension, lack of trust), problematic behavioral processes (e.g., conflict, competition, poor communication), and a lack of shared cognitive states (e.g., unclear roles, lack of shared awareness, competing goals), act as barriers to implementation and are associated with poor implementation outcomes	[40,41,56,61,77,83,86,92,94,95,97 85,]	High confidence	Moderate concerns about coherence and minor concerns about methodology and adequacy do not reduce confidence in this simple descriptive finding
Trust, cohesion, and psychological safety within teams facilitate implementation by contributing to team members’ willingness to speak up and openly share experiences and feedback. Negative affective states, fear of judgment, conflict, and lack of safety hinder implementation	[47–49, 78]	Moderate confidence	A small number of studies and limited diversity in settings and interventions leading to moderate concerns about adequacy and relevance
Open, ongoing, and effective communication within teams facilitates implementation of new practices; poor communication is a barrier	[40, 47, 53, 74]	High confidence	Minor concerns about adequacy do not reduce confidence in this simple descriptive finding
Communication beyond the team facilitates implementation by providing opportunities for team learning	[47, 48, 75]	Low confidence	Variations in definitions and limited data from a small number of studies leading to serious concerns about coherence and adequacy
Poor coordination among healthcare professionals interferes with providing high-quality care and can be a barrier to implementation of new approaches	[40, 95]	Low confidence	Ambiguous findings and thin data in a small number of studies leading to serious concerns about coherence and adequacy and moderate concerns about relevance
Shared goals, mission, and vision within teams facilitate implementation and sustainment	[47, 84]	Low confidence	Only 2 studies in similar settings and only 1 study with adequate data leading to serious concerns about relevance and serious concerns about adequacy

Low quality/low relevance studies not included in synthesis: [43, 46, 51, 52, 60, 63, 64, 66, 69, 71, 73, 76, 79, 82, 87, 90, 93, 96]

#### Overall team functioning

Nine studies examined quantitative associations between overall team functioning and implementation outcomes. Team functioning was positively associated with intervention fidelity in 2 of 3 studies. One study examined implementation of transition programs for adolescents with chronic health conditions in 29 teams. More positive team climate, measured by the Team Climate Inventory (i.e., shared vision, participative safety, task orientation, support for innovation), at study start was associated with greater improvements in quality of chronic care delivery one year later [45]. Additionally, improvements in team climate were associated with greater improvement in care delivery [45]. These findings were consistent across teams working with different patient populations, suggesting the influence of team climate generalizes across teams and settings [45]. Greater team climate for innovation was also associated with greater fidelity (i.e., implementation of more program elements) among DBT teams [47]. In contrast, no significant associations were found between team climate and fidelity to a multifaceted cardiovascular disease management intervention, with qualitative data suggesting variation in the influence of teamwork across practices [77]. There was no overlap in the metrics of association reported in these studies; therefore, we were unable to generate an estimate of the effect of team functioning on fidelity. The quality of the evidence for fidelity was rated very low because of serious methodological limitations, serious inconsistency, and very serious imprecision due to the small number of studies.

Three studies examined associations between teamwork and adoption, with no significant associations found. The first study found that teamwork climate (i.e., perceived quality of collaboration between personnel) was not significantly associated with adoption of a comprehensive safety program in intensive care units, although there were associations between adoption and organizational constructs (e.g., lower safety climate, more management support) [59]. In a study of DBT teams, neither positive nor negative team functioning was associated with the number of DBT modes adopted [72]. The third study assessed relational coordination (i.e., shared goals, communication, respect) in primary care practices implementing patient engagement strategies. Relational coordination was high across practices initially and did not differ for practices with high vs. low adoption, although it increased over time in practices with high adoption [83]. There was no overlap in the metrics of association reported in these studies; therefore, we were unable to generate an estimate of the effect of team functioning on adoption. The quality of the evidence was rated very low because of serious methodological limitations and very serious imprecision due to the small number of studies.

Reach and sustainment were each examined in one quantitative study. DBT teams with more negative functioning had greater reach, suggesting that reach may reflect high workloads; positive functioning was not associated with reach [72]. In VA mental health clinics, team functioning was positively correlated with sustainment of evidence-based trauma-focused psychotherapies and significantly associated with sustainment after controlling for covariates [68]. Two studies examined other implementation outcomes. One found that better team functioning was associated with greater implementation of changes to improve access to care in VA clinics [62]. In the other, primary care practices reporting better teamwork were more likely to be in later stages of transformation to PCMHs than practices with poorer teamwork [88]. Because of the small number of studies examining reach, sustainment, and other implementation outcomes, we were unable to generate estimates of effects or ratings of evidence quality for these outcomes.

Our qualitative review findings are based on 12 studies describing how team functioning influenced implementation processes and outcomes. There was considerable variation across studies in how team functioning was defined and what implementation outcomes were examined. Most findings were based on relatively thin and superficial data. Studies occurred in a variety of healthcare settings with varying resources and implemented diverse interventions. We found with high confidence that 1) Adaptive team functioning, characterized by positive affective states (e.g., trust, mutual respect, belonging), effective behavior processes (e.g., frequent communication and coordination), and shared cognitive states (e.g., clear roles, shared mental models of how to provide care), facilitates implementation and is associated with better implementation outcomes; and 2) Problems in team functioning, including negative affective states (e.g., tension, lack of trust), problematic behavioral processes (e.g., conflict, competition, poor communication), and a lack of shared cognitive states (e.g., unclear roles, lack of shared awareness, competing goals), act as barriers to implementation and are associated with poor implementation outcomes.

#### Affective states

Specific affective states were examined in one quantitative study, three mixed methods studies, and one qualitative study. There was no overlap in the associations between affective states and implementation outcomes reported in quantitative studies (Table 3). In a study of multidisciplinary teams responding to child abuse, affective integration (i.e., liking, trust, respect) was positively associated with acceptability, appropriateness, and feasibility but not time to adoption or reach [65]. In DBT teams, cohesion was associated with fidelity, and qualitative data indicated that liking one’s team members and having a shared team identity were critical to effective implementation [47]. Another study of DBT teams described conflicts and lack of safety and trust within teams resulting in their dissolution [78].

Edmondson and colleagues found that psychological safety and ease of speaking up (i.e., interpersonal climate that allows members to share questions and concerns) were associated with implementation success [48, 49]. In teams with low psychological safety, lower-status team members were hesitant to speak up, hindering change and proficiency in the new practice [49]. Psychological safety was closely related to learning behavior within the team, including speaking up with questions and concerns [48, 49]. From the mixed methods and qualitative studies, we found that trust, cohesion, and psychological safety within teams facilitate implementation by contributing to team members’ willingness to speak up and share experiences and feedback. Negative affective states, fear of judgment, conflict, and lack of safety hinder implementation (moderate confidence).

#### Behavioral processes

Specific behavioral processes, including communication, learning behavior, and coordination, were examined in two quantitative studies, two mixed methods studies, and five qualitative studies. There was no overlap in the associations between behavioral processes and implementation outcomes reported in quantitative studies (Table 3).

Only one study reported quantitative findings for communication. Communication in DBT teams was positively associated with fidelity [47]. Qualitative studies frequently identified communication as a determinant of implementation (Table 4). From qualitative studies, we found that open, ongoing, and effective communication within teams facilitates implementation of new practices; poor communication is a barrier (high confidence).

Quantitative associations between team learning behavior and implementation outcomes were reported in three studies. Team learning behavior in child abuse teams was positively correlated with acceptability and feasibility; it was not associated with appropriateness, time to adoption, or reach [65]. Learning behavior was positively associated with knowledge and use of innovations in nursing teams [91] and with implementation success in surgical teams [48]. Because each of these studies examined different implementation outcomes, we were unable to generate an estimate of the effect of learning behavior or rate evidence quality.

Inter-team communication, specifically speaking up and learning from other teams (i.e., boundary spanning), was identified as a critical part of team learning processes associated with successful implementation [48]. Communication beyond the team was also identified as a facilitator of implementation in two qualitative studies [47, 75]. We found that communication beyond the team facilitates implementation by providing opportunities for team learning (low confidence).

Lastly, two qualitative studies examined coordination among healthcare teams [40, 95]. Findings were somewhat ambiguous and based on thin data. We found with low confidence that poor coordination among healthcare professionals interferes with providing high-quality care and can be a barrier to implementation of new approaches (low confidence).

#### Cognitive states

Specific cognitive states were examined in two quantitative studies. There was no overlap in the associations between cognitive states and implementation outcomes reported (Table 3). The first study found no significant associations between shared goals and implementation outcomes [65]. The second study found that greater team knowledge and skills were associated with greater implementation of key changes to improve access to care; team problem recognition was not associated with implementation [62].

Two studies reported qualitative findings related to shared goals. In VA mental health teams, shared mission differentiated teams with sustained high reach of EBPs from those with low reach [84]. Commitment to a shared goal consistent with the EBP supported sustainment [84]. Similarly, shared goals and vision were identified as a facilitator of DBT programs [47]. We found that shared goals, mission, and vision within teams facilitate implementation and sustainment (low confidence).

#### Summary of team processes/states & implementation outcomes

There was very little overlap in the reported associations between team processes/states and implementation outcomes (Table 3). We were unable to generate estimates of effects for any associations. When there was sufficient overlap to rate evidence quality, the evidence was rated very low quality (Table A2, Additional File 2).

Qualitative review findings are shown in Table 4. We found the following: 1) Adaptive team functioning, characterized by positive affective states (e.g., trust, mutual respect, belonging), effective behavior processes (e.g., frequent communication and coordination), and shared cognitive states (e.g., clear roles, shared mental models of how to provide care), facilitates implementation and is associated with better implementation outcomes (high confidence); 2) Problems in team functioning, including negative affective states (e.g., tension, lack of trust), problematic behavioral processes (e.g., conflict, competition, poor communication), and a lack of shared cognitive states (e.g., unclear roles, lack of shared awareness, competing goals), act as barriers to implementation and are associated with poor implementation outcomes (high confidence); 3) Trust, cohesion, and psychological safety within teams facilitate implementation by contributing to team members’ willingness to speak up and openly share experiences and feedback. Negative affective states, fear of judgment, conflict, and lack of safety hinder implementation (moderate confidence); 4) Open, ongoing, and effective communication within teams facilitates implementation of new practices; poor communication is a barrier (high confidence); 5) Communication beyond the team facilitates implementation by providing opportunities for team learning (low confidence); 6) Poor coordination among healthcare professionals interferes with providing high-quality care and can be a barrier to implementation of new approaches (low confidence); and 7) Shared goals, mission, and vision within teams facilitate implementation and sustainment (low confidence).

### Synthesis: team effectiveness outcomes & implementation outcomes

Team effectiveness outcomes are multidimensional and include performance (i.e., productivity, efficiency, and quality of the team’s work), team viability, and the impact of the team on members’ development [12, 17–19]. Only two studies examined associations between team effectiveness and implementation outcomes. Quantitative findings are presented in Table 5. One quantitative study found that team members’ ratings of team performance were associated with innovation acceptability, appropriateness, and feasibility; performance was not associated with time to adoption or reach [65]. One qualitative study found that positive outcomes for team members (e.g., increased comfort working together, greater knowledge) were associated with sustainment [70]. No studies examined associations of team viability and implementation outcomes.

**Table 5:** Summary of quantitative studies of team effectiveness & implementation outcomes

*Team Effectiveness*	*Implementation Outcome*					
	**Acceptability, appropriateness, feasibility**	**Adoption**	**Fidelity**	**Reach**	**Sustainability**	**Other**
**Team Performance**	***r* = 0.13** (acceptability) ***r* = 0.12** (appropriateness) ***r* = 0.11** (feasibility) [65]	*r* = -0.32, ns [65]		*r* = .18, ns (initial reach) *r* = .34, ns (overall reach) (65)		

Bolded results are statistically significant

*ns* Not statistically significant

#### Summary of team effectiveness outcomes & implementation outcomes

Only one quantitative study examined associations between a dimension of team effectiveness and implementation outcomes (Table 5). Accordingly, we were unable to generate ratings of evidence quality or estimates of any effects. Similarly, because there was only one qualitative study, we were unable to make a review finding.

## Discussion

This systematic review summarizes over 20 years of empirical literature on the associations between teamwork and implementation outcomes in the context of implementation of new practices in health and human services. Consistent with increased attention to teams and reliance on team-based models of care, as well as the growth of implementation science, studies increased substantially over time. We included studies that used quantitative, qualitative, or mixed methods, yielding a total of 58 articles representing 55 studies. Included studies spanned naturalistic implementation evaluations and planned implementation research.

Key findings with high confidence were: 1) Staffing shortages and turnover hinder implementation, 2) Adaptive team functioning, characterized by positive affective states, effective behavior processes, and shared cognitive states, facilitates implementation and is associated with better implementation outcomes. Problems in team functioning, including negative affective states, problematic behavioral processes, and a lack of shared cognitive states, act as barriers to implementation and are associated with poor implementation outcomes, and 3) Open, ongoing, and effective communication within teams facilitates implementation of new practices; poor communication is a barrier. Our results generally align with conventional wisdom and scientific research outside of healthcare, increasing confidence in the findings. Team effectiveness and change management research in other types of organizations and settings (e.g., military, aviation, space exploration) [98–103] is largely converging.

Overall, the literature was heterogeneous, and many studies lacked specificity regarding team composition and implementation activities and outcomes. Teamwork was defined and measured inconsistently and with limited precision across studies, which hindered our ability to draw conclusions about how teams influence implementation processes and outcomes. There was also poor measurement and reporting of implementation outcomes in many studies, consistent with a recent review of research on implementation outcomes [28, 29]. Many studies used broad measures encompassing multiple dimensions of teamwork. Among studies that assessed specific team processes and states, there was very little overlap across constructs assessed. Qualitative studies identified a rich array of specific team processes and states; research to confirm the presence of these factors in other settings and determine their associations with implementation outcomes is needed.

In Table 6, we summarize the limitations of existing research on teams and implementation and provide recommendations for future research. Notably, increased specificity and rigor in how teamwork is conceptualized and assessed is needed to advance our understanding of how teamwork affects implementation processes and outcomes. Limited inclusion of teams and team constructs in implementation theories, models, and frameworks has likely contributed to the neglect of teams in implementation science [20]. Updates to theories, models, and framework should consider integrating teams and team-level constructs [20]. In addition, there are well-established theories of team effectiveness that could inform hypotheses about how specific team constructs affect implementation [104–107].

**Table 6:** Limitations of current research on teams & implementation science and recommendations for future research

Limitations	Recommendations
***Conceptualizing Teams***	
Omission of teams and team constructs from implementation theories, models, and frameworks	Integrate teams and team constructs into implementation theories, models, and frameworks (see 20)
Limited use of theory and research from the science of teams	Use well-established theories of team effectiveness and existing research on teams to develop hypotheses about how specific team constructs will affect implementation processes and outcomes
***Describing Teams***	
Poor definitions of teams. “Team” often used to describe groups of people working in the same setting without describing their interactions	Be clear about whether the group being studied is a team. If it’s a team, what makes it a team? Describe structural characteristics of the team (e.g., size, membership, stability), the purpose of team, and how the team works together (i.e., interdependencies)
Poor reporting of team-level sampling, recruitment, and response rates	Describe sampling processes within and across teams. How were individuals within teams sampled? How were teams sampled? Report team-level response rates for studies with multiple teams
***Assessing Teams & Team Constructs***	
Variations in how teamwork was defined and measured; little consistency across studies	Increase specificity and rigor in how teamwork is conceptualized and assessed Use reliable and valid measures
Limited descriptions of the context within which teams operate	Assess and describe the organizational and system context of teams
***Analyzing Team Data***	
Limited consideration of within-team agreement and justification for aggregation	Evaluate within-team agreement before aggregating to the team level
Frequent use of individual-level data to make inferences about teams (i.e., atomistic fallacy)	Analyze at the team level to draw team-level inferences
Limited consideration of clustering of teams within organizations	When possible, account for clustering of teams within organizations in statistical models
***Interpreting Team Data***	
Limited integration of findings with existing theories and research	Situate findings within the broader literature on teams Use findings to refine implementation theories, models, and frameworks

There is considerable room for improvement in the definition and description of teams and analysis of data from teams. Describing the structure and purpose of teams, as well as interdependencies within the team, can help differentiate teams from groups of individuals who do not constitute a team, an important conceptual distinction that can be difficult to discern in study descriptions. Reporting of sampling and recruitment procedures for teams and team-level response rates is needed. For quantitative studies, use of standardized, validated measures of teamwork constructs is recommended. Researchers should be careful to base inferences about teams on team-level data. Lastly, future research should follow recommendations for improving measurement and reporting of implementation outcomes [29, 108] and consider the multilevel context of teams in theory, measurement, analysis, and interpretation of results [109].

### Limitations

As with all systematic reviews, it is possible that we failed to identify some relevant articles or data. We did not search gray literature or conference abstracts or contact authors for unreported data. Our organization of studies by the IMOI framework is likely imperfect given the broad array of team constructs included and poor reporting in many studies. We included diverse innovations intended to improve patient care, including specific EBPs, clinical practice guidelines, models of care, care bundles, procedural changes, and technological innovations. This diversity in objects of implementation reflects ongoing debates about the necessary strength of evidence for objects of implementation and varying thresholds in different contexts [110]. In this review, high quality studies tended to involve clinical interventions with strong research evidence (e.g., DBT) and clinics in structured and often team-based healthcare systems (e.g., VA). Diversity of innovations and settings provides greater external validity for our findings but may mask some findings specific to certain innovations or settings.

We only included studies of existing teams providing clinical services, however, many studies provided limited descriptions of teams, and in some cases the distinction between clinical teams and implementation/quality improvement teams was unclear. There is increasing attention to use of teams in implementation frameworks [20, 111] and evidence that functioning of implementation teams matters [112, 113]. Research on the composition and functioning of implementation teams is an important area for future research.

## Conclusions

Our systematic review findings indicate that teamwork matters for implementation. However, greater specificity and rigor are needed to advance our understanding of how teamwork influences implementation processes and outcomes. We provide recommendations for improving the conceptualization, description, assessment, analysis, and interpretation of research on teams implementing new practices.

### Supplementary Information

Supplementary Material 1.

Supplementary Material 2.

## Data Availability

All data cited in this review came from published papers and are therefore already available. The data created as part of the review process are included in this published article and its supplementary information files.
