# Supplementary material for: Teamwork and implementation of innovations in healthcare and human service settings: a systematic review
Source: Implement Sci. 2024 Jul 15;19:49. doi: 10.1186/s13012-024-01381-9 (PMC11247800; doi:10.1186/s13012-024-01381-9)
Supplement: Supplementary file 2 — Supplementary Material 2. [file 13012_2024_1381_MOESM2_ESM.docx]

**Additional File 2**

**CERQual & GRADE Evidence Profiles**

Table A1: Evidence Profile: Qualitative Studies of Team Inputs & Implementation Outcomes

Table A2: Evidence Profile: Quantitative Studies of Team Processes/States & Implementation Outcomes

Table A3: Evidence Profile: Qualitative Studies of Team Processes/States & Implementation Outcomes

**Table A1**

*Evidence Profile: Qualitative Studies of Team Inputs & Implementation Outcomes*

| Summary of review finding | Studies contributing | Methodological limitations | Coherence | Adequacy | Relevance | CERQual assessment of confidence in the evidence | Explanation of CERQual assessment |
| --- | --- | --- | --- | --- | --- | --- | --- |
| Dedicated and stable team members facilitate implementation, while instability in team membership is a barrier to implementation. | (49,70,81,94) | Minor methodological limitations.  1 study with results insufficiently substantiated by data. | Minor concerns about coherence.  Some potentially contradictory data. Finding is somewhat oversimplified. | Moderate concerns about adequacy.  Only 4 studies. Less data to support finding about instability. | Moderate concerns about relevance.  2/4 studies focused on new surgical processes; limited diversity in settings and interventions. | Moderate confidence | Only 4 studies with limited diversity in settings and interventions leading to moderate concerns about adequacy and relevance. |
| Staffing shortages and turnover hinder implementation. | (50,67,75,78,92) | Minor methodological limitations.  1 study with inadequate derivation and substantiation of results. | No to very minor concerns about coherence. | Minor concerns about adequacy.  This finding is based on relatively thin and superficial data. However, given that the finding is a simple descriptive finding, there are only minor concerns about adequacy. | No to very minor concerns about relevance. | High confidence | Minor concerns about methodology and adequacy do not reduce confidence in this simple descriptive finding. |
| Team member competency/expertise, experience, and commitment/engagement facilitate implementation. | (40,70,81,84,95) | Minor methodological limitations.  1 study with results insufficiently substantiated by data. | Moderate concerns about coherence.  Studies focused on different team member characteristics and used different definitions. Finding is somewhat oversimplified. | Moderate concerns about adequacy.  Only 5 studies; relatively thin data. | No or very minor concerns about relevance. | Moderate confidence | Only 5 studies with variation across studies and thin data leading to moderate concerns about coherence and adequacy. |
| In multidisciplinary settings, rigid professional roles, hierarchical relationships, and power differentials are barriers to implementation. | (50,53,74,97) | Minor methodological concerns.  1 study with inadequate derivation and substantiation of results. | Moderate concerns about coherence.  Definitions and themes varied across studies. There are plausible alternative explanations. | Moderate concerns about adequacy.  Only 4 studies; relatively thin data. | Minor concerns about relevance.  Limited diversity in settings and interventions. | Moderate confidence | Only 4 studies with variation across studies and thin data leading to moderate concerns about coherence and adequacy. |

**Table A2**

*Evidence Profile: Quantitative Studies of Team Processes/States & Implementation Outcomes*

| Studies Contributing | Limitations/Risk of Bias | Inconsistency | Indirectness | Imprecision | Publication bias | Summary of findings | Evidence quality |
| --- | --- | --- | --- | --- | --- | --- | --- |
| **Fidelity**  3 observational studies  (45, 47, 77) | Serious methodological limitations | Serious inconsistency | No serious indirectness | Very serious imprecision | Strongly suspected | NA | Very Low |
| **Adoption**  3 observational studies  (59, 72, 83) | Serious methodological limitations | No serious inconsistency | No serious indirectness | Very serious imprecision | Strongly suspected | NA | Very Low |

NA: Not applicable (not possible to calculate estimate of effect).

**Table A3**

*Evidence Profile: Qualitative Studies of Team Processes/States & Implementation Outcomes*

| Summary of review finding | Studies contributing | Methodological limitations | Coherence | Adequacy | Relevance | CERQual assessment of confidence in the evidence | Explanation of CERQual assessment |
| --- | --- | --- | --- | --- | --- | --- | --- |
| Adaptive team functioning, characterized by positive affective states (e.g., trust, mutual respect, belonging), effective behavior processes (e.g., frequent communication and coordination), and shared cognitive states (e.g., clear roles, shared mental models of how to provide care), facilitates implementation and is associated with better implementation outcomes.  Problems in team functioning, including negative affective states (e.g., tension, lack of trust), problematic behavioral processes (e.g., conflict, competition, poor communication), and a lack of shared cognitive states (e.g., unclear roles, lack of shared awareness, competing goals), act as barriers to implementation and are associated with poor implementation outcomes. | (40,41,56,61,77,83,  85,86,92,94,95,97) | Minor methodological limitations.  Some studies with results insufficiently substantiated by data. | Moderate concerns about coherence.  Considerable variation in definitions and themes across studies. | Minor concerns about adequacy.  This finding is based on relatively thin and superficial data. However, given that the finding is a simple descriptive finding, there are only minor concerns about adequacy. | No or very minor concerns about relevance. | High confidence | Moderate concerns about coherence and minor concerns about methodology and adequacy do not reduce confidence in this simple descriptive finding. |
| Trust, cohesion, and psychological safety within teams facilitate implementation by contributing to team members’ willingness to speak up and openly share experiences and feedback. Negative affective states, fear of judgment, conflict, and lack of safety hinder implementation. | (47–49,78) | Minor methodological limitations.  1 study with results insufficiently substantiated by data. | Minor concerns about coherence.  Some variation in definitions. Finding is somewhat oversimplified. | Moderate concerns about adequacy.  Only 3 studies, including 1 with inadequate data. | Moderate concerns about relevance.  Only 2 different settings and interventions. | Moderate confidence | A small number of studies and limited diversity in settings and interventions leading to moderate concerns about adequacy and relevance. |
| Open, ongoing, and effective communication within teams facilitates implementation of new practices; poor communication is a barrier. | (40,47,53,74) | No or very minor methodological limitations. | No or very minor concerns about coherence. | Minor concerns about adequacy.  This finding is based on relatively thin and superficial data. However, given that the finding is a simple descriptive finding, there are only minor concerns about adequacy. | No or very minor concerns about relevance. | High confidence | Minor concerns about adequacy do not reduce confidence in this simple descriptive finding. |
| Communication beyond the team may facilitate implementation by providing opportunities for team learning. | (47,48,75) | Minor methodological limitations.  1 study with results insufficiently substantiated by data. | Serious concerns about coherence.  Variations in definitions and vague findings. | Serious concerns about adequacy.  Only 3 studies, and only 1 study with rich data to support explanation. | Minor concerns about relevance.  Diverse settings and interventions but only 3 studies. | Low confidence | Variations in definitions and limited data from a small number of studies leading to serious concerns about coherence and adequacy. |
| Poor coordination among healthcare professionals interferes with providing high-quality care and can be a barrier to implementation of new approaches. | (40,95) | Minor methodological limitations.  1 study with results insufficiently substantiated by data. | Serious concerns about coherence.  Ambiguous and incomplete findings. There are plausible alternative explanations. | Serious concerns about adequacy.  Only 2 studies; thin data. | Moderate concerns about relevance.  Only 2 different settings and interventions. | Low confidence | Ambiguous findings and thin data in a small number of studies leading to serious concerns about coherence and adequacy and moderate concerns about relevance. |
| Shared goals, mission, and vision within teams facilitate implementation and sustainment. | (47,84) | Minor methodological limitations.  1 study with results insufficiently substantiated by data. | Minor concerns about coherence.  Studies examined different implementation outcomes.  Simple descriptive finding | Serious concerns about adequacy.  Only 2 studies; only 1 study with adequate data. | Serious concerns about relevance.  Only 2 studies, both in mental health settings with previously trained providers. | Low confidence | Only 2 studies in similar settings and only 1 study with adequate data leading to serious concerns about relevance and serious concerns about adequacy. |
